# Knowledge and perceptions of air pollution in Ningbo, China

**DOI:** 10.1186/s12889-016-3788-0

**Published:** 2016-11-05

**Authors:** Xujun Qian, Guozhang Xu, Li Li, Yueping Shen, Tianfeng He, Yajun Liang, Zuyao Yang, Wan Wei Zhou, Jiaying Xu

**Affiliations:** 1 Ningbo First Hospital, Ningbo, 315010 China; 2 School of Public Health, Soochow University, #199, Renai Road, Industrial Park District, Suzhou, 215123 China; 3 Ningbo Municipal Center for Disease Control and Prevention, #237, Yongfeng Road, Haishu District, Ningbo, 315010 China; 4 Department of Epidemiology, Capital Institute of Pediatrics, Beijing, China; 5 Division of Epidemiology, JC School of Public Health and Primary Care, The Chinese University of Hong Kong, Shatin, NT, Hong Kong, SAR China; 6 Department of public Health and Tropical Medicine, Tulane University, New Orleans, LA USA

**Keywords:** Haze/ambient air pollution, Health, Knowledge attitudes and practices

## Abstract

**Background:**

The residents’ knowledge, attitudes and practices related to ambient air pollution and health will help to improve the understanding of environmental protection and make environmental health policies more targeted and effective. This study aimed at knowing the attitudes and behaviors towards ambient air pollution and health.

**Methods:**

A cross-sectional survey was conducted in Ningbo, China in January 2015. Personal information and questions pertaining to the knowledge, attitudes and practices towards ambient air pollution and health were collected through questionnaire investigations. Descriptive statistics, chi-square tests and multiple unconditional logistic regression analysis were used.

**Results:**

The questionnaire was completed by 1604 respondents (59.41 % women). The awareness rate was 64.59 % and varied significantly with age, levels of education, and occupation (all *p* < 0.05). Only 5.80 % of the total participants were satisfied with the air quality in Ningbo in 2014. Most respondents (78.80 %) expressed concern about the possible aggravation of the haze. More than 80 % of participants believed that it will take at least 3-5 years or longer before the air quality is improved. Television and internet resources have replaced books and newspapers as the primary sources for obtaining knowledge about haze and related protective measures. 85.22 % of respondents were concerned about air quality index (AQI). Most of the residents have taken protective measures indoors during haze weather. 48.50 % have worn face masks when going outside, the most frequently type of face masks selected were cotton (39.85 %) or gauze face masks (36.24 %). Age and occupation were the main factors associated with the level of knowledge about air pollution (*p* < 0.05).

**Conclusions:**

There were a relatively high knowledge awareness rate, strong health protection consciousness and high enthusiasm for air pollution control among Ningbo residents. The elderly people and less-educated residents are the targetable population for improving environment.

## Background

Ambient air pollution is an increasingly severe public health problem worldwide, especially in China, where environmental deterioration has accompanied rapid economic growth, causing concern both domestically and internationally. The Global Burden of Disease study has confirmed that ambient air pollution is a leading risk factor for adverse health [1–3]. Policies on ambient air pollution have been implemented to monitor China’s serious problem of haze-fog pollution. It will be a long time before the results of these policies are fully known. Valid and reliable estimates of demographic correlates of knowledge, attitudes and practices (KAP) of ambient air pollution on health are essential to support evidence based government policy in this important area [1, 4].

Ningbo, a coastal city located on the eastern coast of China, is relatively developed economically and is one of the busiest port cities in the Yangtze River delta. High levels of haze were experienced for 96 days in 2012, 138 days in 2013 [5]. Ambient air pollution reached “hazardous” levels for eight days in December 2013. Therefore, ambient air pollution in Ningbo is a growing public health problems concern.

A KAP survey is a representative study of a specific population that aims to collect data on what is known, believed and done in relation to a particular topic [6]. It is a crucial element of ambient air pollution control, as little information was available about residents’ KAP of ambient air pollution and health in China. Although several studies have investigated public awareness about ambient air pollution and its adverse health effects in some cities, investigation of Ningbo has not been conducted. This study focused on Ningbo as an example to understand and identify factors associated with the residents’ knowledge of current ambient air pollution, their attitudes toward air pollution control, and the practices of protection in Ningbo. This information could be vital for the local government in regard to the development of more impactful policies and effective measures to reduce air pollution in the future.

## Methods

### Study participants

The household interview was conducted in January 2015. A multistage cluster-sampling method was employed to identify study participants. Generally speaking, there are significant differences between urban and rural area in economic and cultural level in China. To make the study more representative, the sampling frame used in the first stage defines primary sampling units (PSU) as either districts that represent urban or counties that represent rural areas. Then one urban district (Jiangbei) and one rural county (Ninghai) were randomly selected respectively from six districts and five counties of Ningbo. In the second-stage sampling units (SSU), two residential streets in each of the selected district and county were identified as the sample areas. Formula for estimating sample size as follows: $N= deff\times \frac{1.96^2\widehat{p}\widehat{q}}{d^2}$, where deff is the design effect, $\widehat{p}$ is the estimator of a proportion, *p* = 0.5,q = 0.5.d = 0.1,

$$ N=2\times \frac{1.96^2(0.5)(0.5)}{0.1^2}=192.08=193 $$

in the study, we increased the minimum sample size by 10% to make up for the lack of questionnaire design. Hence, the final sample size for each residential street was 212 households. There were four residential streets in the study, so 848 households were selected. In each household family members aged 14 to 65 years, registered permanent ningbo residence (living at their present residence for more than one year in Ningbo before this survey) were screened undergo the interview. Overall, 1680 participants were approached, of whom 1604 responded (responserate:95.47 %).

### Questionnaire investigation

We conducted the survey in a face-to-face interview style to assess the residents’ KAP with regard to the effects of haze-fog pollution on health. The validity of the questionnaire was established through content and expert validity, The internal consistency of knowledge outcomes were tested by Cronbach’s alpha. A 24-item questionnaire designed by public health experts was used, focusing primarily on three kinds of questions: demographic characteristics (e.g., age, gender, educational level, residence, average annual household income, occupation, etc.), knowledge (e.g., The question was “what health problems do you think are brought about by air pollution?”. The response alternatives were “lung cancer”,“difficulty breathing”, “heart problems” or “I’m not sure”.) and attitudes (e.g., The question was “Were you satisfied with the air quality in Ningbo last year?”. The response alternatives were “yes”,“no” or “I do not care about this problem at all”) towards ambient air pollution, and behavior related to the ambient air pollution. There contains five questions about knowledge related to the haze and heath. The scores varied from 0–5 points, each right answer was given a score of 1 and the wrong answer was given a score of 0. Residents who gave the right answer to all questions were classified into the “known” level and those who failed to any one question were classified into the “unknown” level. We defined the awareness rate of knowledge as the percentage of respondents who were classified into the “known” level.

### Data analysis

Epidata 3.02 was applied to establish a database, and double data entry was used to reduce errors. SAS 9.2 was used for statistical analyses. Chi-square tests were applied for demographic factors including respondents’ gender, age groups, educational level and occupation. Multiple unconditional logistic regression was used to assess the associations between knowledge outcome and demographic factors, with adjusted odds ratios (ORs) and 95 % confidence intervals (CIs) being presented. *p* < 0.05 was considered as statistically significant.

## Results

Of the 1604 subjects, 40.59 % were males and 59.41 % were females. The mean age of respondents was 39.31 years (range from 14 to 65 years) with a standard deviation of 14.36 years. Table 1 shows the general demographic characteristics.

**Table 1:** Demographic characteristics of the interviewees (*n* = 1604)

Characteristics	Frequency (n)	Percentage (%)	Frequency of awareness (n)	Awareness rate (%)	*χ* ^2^	*P* value
Districts						
Jiangbei Districts	852	53.11	599	70.31	25.96	<0.0001
Ninghai County	752	46.89	437	58.11		
Gender						
Male	651	40.59	413	63.44	0.63	0.4270
Female	953	59.41	623	65.37		
Age (years)						
14-	478	29.80	362	75.73	111.33	<.0001
30-	411	25.62	297	72.26		
40-	249	15.52	165	66.27		
50-	271	16.90	121	44.65		
≥ 60	195	12.16	91	46.67		
Education						
≥ College	755	47.07	561	74.30	100.78	<.0001
High school	355	22.13	242	68.17		
Junior high school	313	19.51	155	49.52		
≤ Elementary school	181	11.28	78	43.09		
Occupation						
Manual worker	141	8.79	78	55.32	128.72	<.0001
Homemaker and retired	350	21.82	147	42.00		
Student	182	11.35	149	81.87		
Cadre and technician	583	36.35	426	73.07		
Service	287	17.89	199	69.34		
Unemployed	61	3.80	37	60.66		

The Cronbach’s alpha values for testing the internal consistency of knowledge outcomes were between 0.655 and 0.827. The awareness rate in respect of the haze and its adverse effects on health was 64.59 %(1036/1604). There was statistically significant difference in the awareness between urban and rural areas as well as age groups (*p* < 0.05). In addition, awareness declined significantly with increasing age, but increased significantly with the increasing levels of education (*p* < 0.05). There was also significant difference among occupation groups, with students demonstrating the highest level of awareness, accounting for 81.87 % (*p* < 0.05). But no statistically significant difference was found in the awareness between men and women (*p* > 0.05) (Table 1).

### Attitudes

Of those 1604 residents, only 5.80 % were satisfied with the air quality in Ningbo in 2014. The majority of residents paid attention to ambient air pollution, accounting for 78.24 %. 21.13 % of residents believed the rate of the haze in Ningbo was “high”. To the possible aggravation of the haze, 78.80 % of the residents expressed that they “worry about”, while 16.34 % expressed “do not worry about”. 15.96 % of residents thought that the haze will improve in the short-term, 57.92 % of the residents believe that the haze will improve within 3-5 years, while 26.12 % of the residents believed the haze will take at least 10 years to improve. 45.08 % of the respondents agreed that the government should take primary responsibility for the haze, while 42.83 % believed that every citizen should take primary responsibility for improving the haze. The sources of haze information most commonly reported in this study were from television and internet resources, accounting for 57.42 %, while 22.88 % of the residents from books and newspapers, and 19.70 % from expert lecture and friends (Table 2).

**Table 2:** The attitudes of the haze and related health risk

Survey content	N	%
Were you satisfied with the air quality in Ningbo last year?		
Yes	93	5.80
No	641	39.96
Average	870	54.24
Have you paied attention to the haze in the community where you live?		
Yes	1255	78.24
No	68	4.24
Indifferent	281	17.52
How severe would you say is the haze in the community where you live?		
Low	332	20.70
Moderate	933	58.17
High	339	21.13
How would you feel about the possible aggravation of the haze?		
Worry about	1264	78.80
Do not worry about	262	16.34
No concern of mine	78	4.86
How long do you think it will take for air quality to improve?		
In the short-term	256	15.96
Within 3-5 years	929	57.92
At least 10 years	419	26.12
Whom do you think should be primary responsible for the haze?		
The government	723	45.08
Every citizen	687	42.83
The industries	194	12.09
What is your favorites way to obtain knowledge refer to the haze and related protective measures?		
Television and internet	921	57.42
Books and newspapers	367	22.88
Expert lecture and friends	316	19.70

### Practices

The percentage of residents’ practices taken during hazy conditions is summarized in Table 3. Among the 1604 residents, 66.77 % (1071/1064) of them have taken protective measures indoors, and most of them have taken measures such as using air purifiers, putting up green plants, using activatedcarbon and so on. 74.32 % (1192/1604) have reduced or eliminated outdoor exercise. 67.33 % (1080/1604) have cut down travel during weekends. 66.46 % (1066/1604) have reduced times of opening windows to air the room. 85.22 % (1367/1604) were concerned about air quality index (AQI). When be asked whether wearing face masks or not when going outside, half of them (51.50 %,826/1604) answered ‘no’. And another part of them, 48.50 % (778/1604) have worn face masks, the most frequently type of face masks selected by them were cotton (39.85 %,310/778) or gauze face masks (36.24 %,282/778). Minor of them have chosen N95 face masks (8.10 %,63/778).

**Table 3:** Percentage of residents’ practices taken in haze weather

Survey content	N	%
Related protective measures taken indoors		
Yes	1071	66.77
No	533	33.23
What related protective measures have you taken indoors? (multiple choice)		
Use of air purifiers	342	16.43
Putting green plants	827	47.90
Putting activated carbon	281	26.24
Others	101	9.43
Reduce or eliminated outdoor exercise		
Yes	1192	74.32
No	412	25.68
Cut down travel during weekend		
Yes	1080	67.33
No	524	32.67
Reduce times of opening windows to air the room		
Yes	1066	66.46
No	538	33.54
Concern or learn about air quality index		
Yes	1367	85.22
No	237	14.78
Wear face masks when going outside		
Yes	778	48.50
No	826	51.50
Which type of face masks do you used?		
Cotton face masks	310	39.85
Gauze face masks	282	36.24
N95 face masks	63	8.10
others	123	15.81

### Variables associated with awareness rate

The demographic factors associated with awareness rate used multiple unconditional logistic regression analysis are shown in Table 4. Two variables had a significant influence on knowledge awareness rate: age and occupation. Taking “age 14-29” as the baseline group, the knowledge awareness rate of age 30–39, 40–49, 50–59, and ≥ 60 groups were lower, In other words, those who were 14-29 years old were the most likely to have a high level of knowledge. Assigning the highest knowledge awareness rate occupation group “Student” as the baseline group, the knowledge awareness rate of other groups were lower.

**Table 4:** Odd ratios and confidence interval of variables associated with awareness rate in multiple unconditional logistic regression analysis

Characteristics	β	OR	OR 95 % CI^a^	*P* value
Age group				
14-29(reference)		1	0.00-0.00	
30-39(1)	0.18	1.19	0.88-1.60	0.26
40-49(2)	0.45	1.58	1.13-2.20	<0.01
50-59(3)	1.34	3.84	2.79-5.27	<0.01
60-(4)	1.26	3.54	2.49-5.02	<0.01
Occupation group				
Student(reference)		1	0.00-0.00	
Cadre and technician(1)	0.48	1.62	1.07-2.45	<0.05
Service(2)	0.66	1.94	1.24-3.04	<0.01
Unemployed(3)	1.04	2.84	1.51-5.36	<0.01
Manual worker(4)	1.26	3.54	2.15-5.83	<0.01
Homemaker and retired(5)	1.80	6.05	3.94-9.29	<0.01

^a^ *CI* confidence interval, *OR* odds ratio. Note: assigned “the score of knowledge ≥ 5” to 1, others to 0; Assigned “age 14-29” to 0 (reference group), “30–39” = 1, “40–49” = 2, “50–59” = 3, “≥60” = 4; Assigned “occupation group Student” to 0(reference group), “Cadre and technician” = 1,“Service” = 2; “Unemployed” = 3, “Manual worker” = 4,“Homemaker and retired” = 5

## Discussion

Previous studies have shown low knowledge awareness rate (27. 9 %) about particulate matter that has an aerodynamic diameter of 2.5 microns or smaller (PM_2.5_) in Guangzhou residents [7]. But in this survey, Ningbo residents showed a higher awareness level at the haze and its adverse impact on health despite experiencing a lower level of exposure to ambient air pollution in China. It indicates that Ningbo residents have a certain understanding on hazy-fog pollution and its adverse impact on health. This is primarily due to Ningbo suffering from widespread heavy smog and hazardous levels of particle pollution twice in 2013 [5], which enabled the local residents to have a better understanding of ambient air pollution. Even the elderly have good understanding of the professional vocabulary like PM_2.5_. In addition, rising standards of living also contributes to the demand for improved air quality. And what should be acknowledged is that the differences between the known and unknown levels definition about the haze and its adverse impact on health might affect the awareness rate.

Although not many residents were satisfied with the air quality in Ningbo in 2014, only 21.13 % of residents believed the haze in Ningbo was “serious”. This is mainly because Ningbo’s air quality is relatively good compared with other major China cities. Ningbo’s environmental air quality have been ranked 15th according to the air quality comprehensive index among the first 74 key cities executing the new air quality standards, and have been the 4th among the 25 major cities located in the Yangtze River Delta, better than Shanghai, Nanjing, Hangzhou and Suzhou in 2014 [8]. According to the related reference, the annual average concentration of PM_2.5_ was 46 μg/m^3^ in Ningbo in 2014, which was much lower than that in cities such as Beijing (72.6 μg/m^3^), Xi’an (78 μg/m^3^) [9]. Although rarely will the city be shrouded by the extreme haze weather, the issues involved with healthy air quality grow year by year. Scince People intuitively feel the change, the majority of residents paied attention to the haze in the community where they live and worried about the possible aggravation of the haze. But it is inspiring to see that 57.92 % of residents considered the air pollution be improved within 3-5 years, they also felt that both the government and individuals have an equal responsibility to improve the air quality. This demonstrates that the residents express a strong desire to engage, support and participate in the activity of governing the haze-fog pollution. Television and the internet are replacing traditional newspapers and expert lecture as the most common ways to obtain knowledge about ambient air pollution, a result consistent with findings reported in the survey in Beijing [10]. This illustrates changes in the way information is disseminated with the development of society. Residents are more inclined to use more visual, auditory and intuitive ways to obtain information, suggesting government meet various kinds of demands for residents such as resources in local dialect for the elderly and simplified materials for less educated people in order to improve the effect of environmental health publicity and education.

In this study, during haze weather, most of the residents have taken some protective measures indoors, such as using air purifiers, putting up green plants, using activated carbon and so on. Reducing outdoor exercise and weekend travel, learning about AQI and wearing face masks were also important measures for the residents to protect themselves from the haze. All these practices have indicated that certain self protection consciousness seems to be developing. But on the choice of the face masks, the most frequently type of face masks selected by those residents were cotton or gauze face masks that used to break the wind rather than anti-smog face masks that offer protective effects. According to some studies, the cotton and gauze face masks provide poor protection against the haze. [11–13]. Epidemiological studies demonstrate that no lower “safe” threshold of exposure seems to exist at the population level [14, 15]. The European Commission (EC) acknowledges that the current standards are insufficient for the protection of public health, particularly in reference to the World Health Organization (WHO) air quality guidelines [16]. Moreover, levels of air pollution remain high and are increasing in China [17]. Therefore interventions which separate people from pollution as components of formal strategies are of great importance. However, they have been largely overlooked according to an international multidisciplinary workshop [18]. Evidence has shown that interventions can reduce air pollutants exposure, especially measures such as air filtration, closing windows and air conditioning, using a particulate respirator face mask. These measures are shown to be feasible and effective [19]. Therefore, strategic priorities should go beyond reducing national level of outdoor ambient concentrations. They should address the effects of specific policy as well as the protective role of masks and air purifiers.

Univariate and multivariate logistic regression analysis in this study showed a correlation between residents’ age, occupation, educational level and knowledge awareness rate of air pollution. In this study those who were more educated were associated with higher awareness level and were more likely to pay attention to the negative health effects from air pollution exposure, which was consistent with most previous studies [20–23]. On the other hand, a study had shown no significant associations between education level and high degree of concern with air pollution [24], but a study from the United States indicated individuals with lower income and educational attainment appear to have been more perceptive and responsive to air quality [25]. Thus, further studies are needed to clarify these issues.

Controversy exists with regards to the association of the knowledge awareness rate with age, this study indicated that the awareness rate of haze in the youth was significantly higher than that in the elderly. There are studies that have not found a significant association between age and perceptions of air quality [26, 27]. Different connections observed in another study found that young respondents pay more attention to environmental issues while the elderly emphasize health and safety [28]. Other studies found that the youth had poor awareness rate compared to the elderly [10, 22]. Most of the studies have not given a clear explanation for these differences. Further research needs to investigate the interaction between age and awareness rate of air pollution.

When comparing the residents’ occupations, in this study, the retired and the manual laborer showed a lower degree of concern and awareness rate than those groups like the student, the cadre and technician. This may be due to influencing factors of education level, economical status and social status.

The relationship between age, education and awareness rate is controversial, recent studies could not provide a clear explanation for these differences. This could be due to a lack of a uniform assessment criteria for knowledge awareness rate, the different control of age of research subjects. Thus, further research is needed to help understand factors shaping people’s awareness.

## Conclusions

Although Ningbo residents are exposed to relatively lower level ambient air pollution than the rest of the nation, it showed a large percentage of residents are aware of the haze problem and the correlation between air pollution and health. However, knowledge was still relatively low in some groups, such as the less educated, the elderly and some type of occupation. This may help policymakers make targeted advocacy and guidance to make residents aware of sources of air pollution and related health risks.

## Abbreviations

AQI: Air quality index
CI: Confidence interval
KAP: Knowledge, attitudes and practices
OR: Odds ratio
PM_2.5_: Particulate matter that has an aerodynamic diameter of 2.5 microns or smaller
WHO: World Health Organization
